# Engineering essential genes with a “jump board” strategy using CRISPR/Cas9

**DOI:** 10.17912/micropub.biology.000315

**Published:** 2020-10-08

**Authors:** Ye Duan, Sungwook Choi, Charles Nelson, Victor Ambros

**Affiliations:** 1 University of Massachusetts Medical School, Worcester, MA 01605, USA

**Figure 1:**
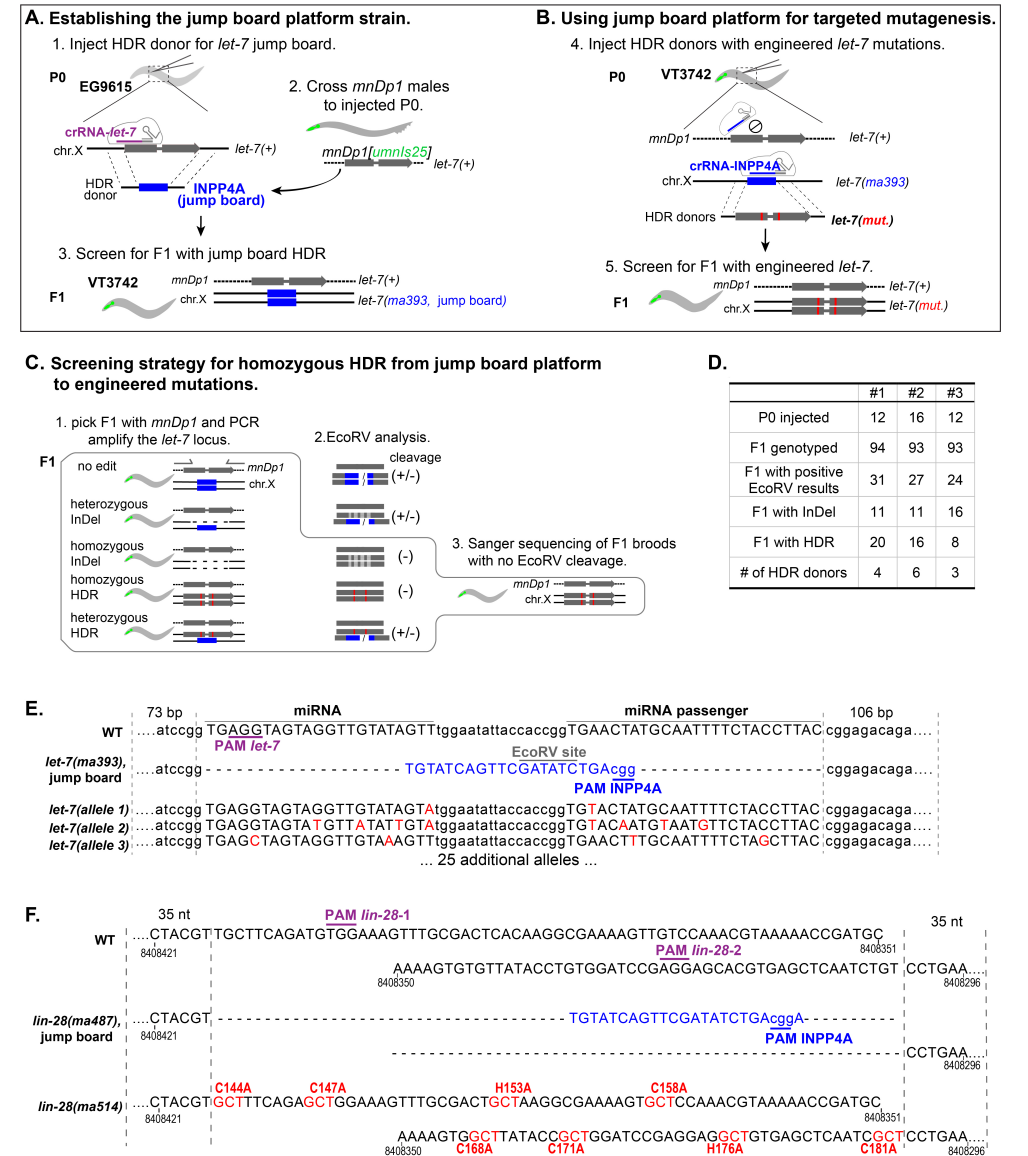
**A-B.** Schematic diagrams of the jump board strategy for engineering the *let-7* locus. To generate the jump board strain (**A**), hermaphrodites of the strain EG9615*(oxSi1091[mex-5p::Cas9(smu-2 introns) unc-119(+)] II; unc-119(ed3) III)* carrying a transgene expressing the Cas9 protein were injected with a crRNA targeting *let-7* (purple), tracrRNA and dsDNA HDR donor to replace the 64 bp sequence corresponding to the precursor-*let-7* transcript with the 23 bp jump board sequence (blue). To permit recovery of F1 progeny carrying a lethal jump board *let-7* null allele, injected P0 hermaphrodites were crossed to males carrying the *mnDp1 [umnIs25]* genetic balancer, which contains a wild type *let-7* locus, as well as an integrated *myo-2p::GFP* transgene. The resulting jump board strain, VT3742(*oxSi1091 II; mnDp1[umnIs25] (X;V)/+ V; let-7(ma393) X*) was used as a platform for repeated mutagenesis at the *let-7* locus. To use the established jump board strain for mutagenesis (**B**), the *let-7* jump board allele *(ma393)* in VT3742 was targeted by the universal jump board guide INPP4A-crRNA and replaced by HDR with multiplexed dsDNA donors containing the *let-7* locus with engineered mutations (red). The wild type allele of *let-7* contained on *mnDp1* is resistant to editing, since INPP4A-crRNA is directed to the jump board sequence, permitting the recovery of lethal *let-7* mutations complemented by *mnDp1*. **C**. Screening strategy for (**B**) using EcoRV restriction analysis. After injection of VT3742, F1 animals with *mnDp1* balancer (detected by pharyngeal GFP) were collected after egg laying and subjected to PCR with flanking primers, and the PCR products were digested by EcoRV. For F1 broods with no EcoRV cleavage (homozygous HDR or homozygous InDel), the PCR products were subjected to Sanger sequencing to identify homozygous HDR lines. Note that heterozygous HDR can also be identified by genotyping the F2 without *mnDp1* followed by Sanger sequencing.However, we have consistently observed that most F1 animals carrying HDR products were homozygous for the HDR alleles. **D.** Summary of three mutageneses of *let-7* locus using the jump board platform. Note that more F1 animals with co-CRISPR phenotypes were isolated than were genotyped. **E**. Aligned sequences of wild type *let-7*, *let-7(ma393)*, and examples of *let-7* alleles generated by replacement of jump board. Color schemes are consistent with (**A-B**). **F**. Aligned sequences of the zinc finger region of *lin-28* representing the application of jump board strategy for protein coding gene. The starting strain VT3711(*lin-28(ma416) I; maIs105 V*) contains a *lin-28* locus (*ma416*) expressing functional LIN-28 protein tagged with 3xFLAG::6xHIS. The endogenous zinc finger domain (C144-C181, 114 bp) of *lin-28* was targeted by two crRNAs in Cas9 RNP and replaced with the jump board sequence using a ssDNA HDR donor. The resulting *lin-28* jump boardallele *lin-28(ma416ma487)* had severe heterochronic phenotypes (including sterility) and was then crossed with *lin-46(ma164)*, a genetic suppressor of *lin-28(lf),* to produce the jump board targeting strain, VT3914(*lin-28(ma416ma487) I; lin-46(ma164) V*) (Pepper *et al.*, 2004). The *lin-28* jump board allele (*ma487)* was subsequently targeted by INPP4A-crRNA and a dsDNA HR donor with 35/35 bp homology to replace *ma487* with a *lin-28* locus carrying a compound mutation (*ma514*) in which 8 critical amino acid residues of the two zinc fingers were replaced by alanine residues. The numbers indicate the positions at chr. I according to Wormbase *WS277*.

## Description

The application of CRISPR/Cas9 genome editing has revolutionized the genetic analysis of gene function *in vivo* by enabling highly efficient and precise *in loco* mutagenesis of defined genomic loci (Adli, 2018). In particular, CRISPR/Cas9 mediated homologous directed recombination (HDR) permits the introduction of complex genetic modifications at single nucleotide resolution (Cong *et al.*, 2013; Dickinson *et al.*, 2013). However, the targeted mutagenesis of essential genes using CRISPR/Cas9 can be challenging since the recovery of deleterious mutations of an essential gene may require a genetic balancer with a wild type copy of the gene (or alternatively a genetic suppressor locus) that can be later outcrossed for phenotypic analysis of novel targeted mutations. Genetic balancers are commonly available for model organisms such as worms or flies, but in the context of CRISPR/Cas9 genome editing, the balancer itself can be edited along with the targeted genomic locus, dramatically reduce the efficiency of recovering the desired loss of function alleles. Therefore, editing of essential genes would be facilitated by a strategy where the targeted genomic locus is efficiently edited, whilst the balancer locus is resistant to editing.

Here, we describe a platformed “jump board” strategy and its application in systematically engineering the essential microRNA *let-7* (Fig. 1A-E) and protein coding gene *lin-28* (Fig. 1F) in *C. elegans*. We chose the jump board protospacer sequence (INPP4A) which is (1) comprised of a PAM site and a protospacer antisense to a crRNA with experimentally confirmed high editing efficiency (INPP4A-crRNA), and (2) non-homologous to *C. elegans* genome, including the genetic balancer we used (*mnDp1)*. Notably, the jump board protospacer contains an EcoRV restriction site, which can be utilized for rapid large-scale genotyping by which HDR events can be identified in the F1 generation (Fig. 1C). Using the jump board strategy, we have so far created 28 *let-7* alleles for various experimental purposes, among which 15 alleles showed lethality and require rescue by *mnDp1*. Note that the *let-7* jump board allele (*ma393*) itself is a new *let-7 null* allele in which the precursor-*let-7* is completely removed.

We have also used the jump board strategy to mutate the two CCHC motifs at the zinc finger domain of *lin-28* (Fig. 1F) (Mayr and Heinemann, 2013; Van Wynsberghe *et al.*, 2011). *lin-28(lf)* phenotypes include near-sterility, which could have been mitigated by using a genetic balancer for chromosome I (analogous to the approach for *let-7*), but in this case we employed a *lin-46(lf)* genetic background which suppresses *lin-28(lf)* phenotypes (Pepper *et al.*, 2004). We suggest that *lin-28* represents an example of how the jump board strategy offers significant advantages for systematic mutagenesis of protein coding genes, as well as microRNAs or other non-coding sequences.

The jump board strategy described here can facilitate the targeted mutagenesis at essential genes for a number of reasons: 1) for genetic loci that lack convenient and efficient protospacer sites, the jump board provides a highly efficient foreign crRNA/protospacer platform; 2) the jump board protospacer sequence contains a convenient restriction site that facilitates rapid large-scale genotyping; 3) the targeting of a foreign sequence protospacer enables the use of genetic balancers unconfounded by undesired editing of the balancers. Conventionally, essential genes can also be edited by targeting the wild type locus, and employing a balancer to rescue the mutations, either by crossing the P0s with balancer-carrying males, or by including the balancer from the outset. In the first instance, the jump board strategy eliminates the need to cross the P0s, and in the second instance, the fact that only the jump board sequence is targeted eliminates the complications associated with unwanted editing of the balancer, which include loss of homozygous edited F1s, and extra crossing step and/or genotyping to distinguish balancer edits from chromosomal edits. We thus suggest that the jump board strategy, although requiring an extra step to establish the platform, offers particular advantages for repeated mutagenesis of individual loci of interest, where throughput is promoted by the highly efficient INPP4A protospacer and the ease of screening for desired edits. We also note that the jump board strategy also prevents cleavage of dsDNA HDR donor molecules or re-cleavage of converted loci, both of which can impact HDR efficiency. Finally, *C. elegans* gene editing technology using CRISPR continues to improve through innovation, for example, to the design of donors and the compositions and preparation of reagent mixtures (Dokshin *et al.*, 2018; Ghanta and Mello, 2020; Richardson *et al.*, 2016; Song *et al.*, 2016). These improvements can be applied to the jump board HDR approach for even greater throughput of targeted mutagenesis.

## Methods

**Genome editing at the *let-7* genomic locus**

Sequences corresponding to the WT precursor-*let-7* and its 500 bp flanking regions were cloned into pCR2.1-TOPO vector. The Q5 mutagenesis kit (NEB, Cat:E0554) was used to generate the mutant plasmids. dsDNA donors were generated from the mutagenized plasmids by PCR with 73/106 bp flanking the precursor-*let-7* and purified by ethanol precipitation. Injection mixtures containing final concentrations of 30 ng/µl AltR_Cas-9_crRNA_let-7 (step 1) or AltR_Cas-9_crRNA_INPP4A (step 2) (PAM sites shown in Fig. 1D), 10 ng/µl AltR_Cas-9_crRNA_dpy-10_cn64 as co-CRISPR marker (Arribere *et al.*, 2014), 75 ng/µl Alt-R tracrRNA (IDT, Cat:1072532), 10 ng/µl each dsDNA donor and 1X duplex buffer (IDT, Cat: 11010301) were incubated at room temperature for 10 min for pre-annealing and injected into the gonad of young adults of strain EG9615*(oxSi1091[mex-5p::Cas-9(smu-2 introns) unc-119(+)] II; unc-119(ed3) III)* (step 1) or VT3742(*oxSi1091 II; mnDp1[umnIs25] (X;V)/+ V; let-7(ma393) X*) (step 2). We suggest injecting 4-8 P0 for single HDR donor and 8-16 P0 for multiplexed HDR donors.

**Genotyping the products of HDR at the *let-7* locus**

For both steps (Fig. 1A and B), F1 dumpy/roller animals with *mnDp1[umnIs25]* (detected by pharyngeal GFP) were picked and genotyped by PCR after egg laying, and the PCR products were analyzed by restriction digestion using EcoRV (NEB, Cat:R3195S) at 1 unit/µl PCR product for 30 min. In the first step (Fig. 1A), samples from the broods with jump boardHDR should have EcoRV cleavage. In the second step (Fig. 1B), samples containing either homozygous HDR or homozygous InDel should have no EcoRV cleavage. Samples with candidate HDR (with EcoRV cleavage in Fig. 1A and with no EcoRV cleavage in Fig. 1B) were subjected to Sanger sequencing, and F1 broods with homozygous HDR were selected and outcrossed. For the genotype screen at the second step, we suggest screening 48 F1 for single HDR donor and 96 F1 for multiplexed HDR donors.

**Genome editing and genotyping at the *lin-28* genomic locus**

In the first step of editing, the Ultramer single-strand DNA donor with 35/35 nt flanking homology was obtained from IDT. The injection mixture containing final concentrations of 15.3 ng/µl AltR_Cas-9_crRNA_lin-28_1, 15.1 ng/µl AltR_Cas-9_crRNA_lin-28_2 (PAM sites shown in Fig. 1F), 7.2 ng/µl AltR_Cas-9_crRNA_dpy-10_cn64, 82.6 ng/µl Alt-R tracrRNA, 159.1 ng/µl ssDNA donor, 0.57 ng/µl AltR-S.p.Cas9 nuclease (IDT, Cat: 1081058) and 1X duplex buffer were incubated at 4 °C for 15 min for pre-annealing and injected into VT3711(*lin-28(ma416) I; maIs105 V*)at young adult stage. F1 dumpy animals were picked and genotyped by PCR (after egg laying) using flanking primers, and samples with PCR products of the expected (reduced) size were confirmed by Sanger sequencing.

In the second step of editing, dsDNA HDR donors were generated with 35/35 bp flanking homology following a protocol analogous to that for the *let-7* locus. Injection mixture containing final concentrations of 30.7 ng/µl AltR_Cas-9_crRNA_INPP4A, 7.2 ng/µl AltR_Cas-9_crRNA_dpy-10_cn64, 82.6 ng/µl Alt-R tracrRNA, 108 ng/µl dsDNA donor, 0.57 ng/µl AltR-S.p.Cas9 nuclease and 1X duplex buffer were incubated at 4 °C for 15 min for pre-annealing and injected into the gonad of VT3914(*lin-28(ma416ma487) I; lin-46(ma164) V*)at the young adult stage. Genotyping was performed using PCR, EcoRV digestion, and Sanger sequencing, as described above.

## Reagents

VT3742(*oxSi1091 II; mnDp1[umnIs25] (X;V)/+ V; let-7(ma393) X*), VT3914(*lin-28(ma416ma487) I;lin-46(ma164) V*) strains and oligo sequences are available upon request.
